# Real-world evaluation of effectiveness, persistence, and usage patterns of monotherapy and combination therapy tofacitinib in treatment of rheumatoid arthritis in Australia

**DOI:** 10.1007/s10067-021-05853-x

**Published:** 2021-08-09

**Authors:** Paul Bird, Geoffrey Littlejohn, Belinda Butcher, Tegan Smith, Catherine O’Sullivan, David Witcombe, Hedley Griffiths

**Affiliations:** 1 University of New South Wales, Kensington, New South Wales Australia; 2 OPAL Rheumatology Ltd, Sydney, New South Wales Australia; 3 Monash University, Clayton, Victoria Australia; 4 WriteSource Medical Pty Ltd, Lane Cove, New South Wales Australia; 5 Pfizer Australia, Sydney, New South Wales Australia; 6 Barwon Rheumatology Service, Geelong, Victoria Australia

**Keywords:** Real-world, Rheumatoid arthritis, TNF-inhibitors, Tofacitinib, Treatment persistence

## Abstract

**Objective:**

This study aimed to describe the real-world effectiveness and treatment persistence among patients with rheumatoid arthritis treated with monotherapy and combination therapy tofacitinib and biologic disease-modifying antirheumatic drugs (bDMARDs).

**Methods:**

This was a post hoc analysis of a retrospective, non-interventional study that extracted data for patients treated with tofacitinib or bDMARDs from the Australian OPAL dataset between March 2015 and September 2018. Monotherapy tofacitinib and bDMARDs and combination therapy tofactinib and bDMARDs were propensity score matched and treatment effectiveness and persistence of the groups were evaluated.

**Results:**

In the bDMARD and tofacitinib monotherapy and combination therapy matched populations there were 1300 bDMARD initiators (*n* = 564 monotherapy) and 650 tofacitinib initiators (*n* = 282 monotherapy). In the bDMARD and tofacitinib monotherapy matched groups, 62.9% and 66.7% were in DAS-28 CRP disease remission after 18 months of treatment, respectively. In the combination therapy bDMARD and tofacitinib groups, 50% and 58.9% were in DAS-28 CRP disease remission after 18 months, respectively. The median treatment persistence was similar between the monotherapy bDMARD and tofacitinib treatment groups (36.7 months (95% CI 27.4 to “not reached’) and 34.2 months (95%CI 30.3 to “not reached”) respectively) as well as the combination therapy bDMARD and tofacitinib groups (32.2 months (95% CI 25.7 to 34.4) and 32.7 months (95%CI 28.7 to “not reached”, respectively).

**Conclusions:**

Patients receiving combination therapy with tofacitinib or bDMARDs had higher disease activity scores at index than patients receiving monotherapy. Monotherapy with tofacitinib or bDMARDs, and combination therapy with tofacitinib or bDMARDs demonstrated similar treatment effectiveness and persistence, respectively.

**Table:** 

**Key Points** • *This study provides real-world evidence regarding effectiveness, treatment persistence, and treatment patterns, among patients with rheumatoid arthritis (RA) treated with monotherapy or combination therapy tofacitinib.* • *The study suggests that monotherapy and combination therapy tofacitinib is an effective intervention in RA with persistence and effectiveness comparable to bDMARDs.*

**Supplementary Information:**

The online version contains supplementary material available at 10.1007/s10067-021-05853-x.

## Introduction

Rheumatoid arthritis (RA) is a systemic autoimmune disease characterized by chronic inflammation of the synovial tissue and destruction of the adjacent cartilage and bone [1]. The exact prevalence is unknown, but it is estimated to affect 0.3–1.2% of the population [2]. As no cure exists, the main goal of treatment is to manage the patients symptoms and preserve function by controlling inflammation and preventing progressive structural damage [3]. This is achieved through treatment with various combinations of therapies including analgesics, corticosteroids, and synthetic or biologic disease-modifying antirheumatic drugs (DMARDs). Physical, occupational, and psychological therapy as well surgery can also play an important role in a patient’s ongoing management.

In Australia, the cost of biologic or targeted synthetic DMARDs (b/tsDMARDs) for the treatment of RA is subsidized if the patient has documented high levels of clinical and laboratory disease activity and has not responded to a pre-specified combination of conventional synthetic DMARDs (csDMARDs), including at least two of methotrexate, hydroxychloroquine, sulfasalazine, or leflunomide [4]. The b/tsDMARD therapies available in Australia include the tumor necrosis factor inhibitors (TNFi) adalimumab, etanercept, certolizumab pegol, golimumab, and infliximab, the interleukin 6 inhibitor (IL6i); tocilizumab, the cytotoxic T-lymphocyte antigen 4 modified antibody; abatacept, the anti-CD20 monoclonal antibody; rituximab, and the more recent mode of action (MOA) therapy, the Janus kinase inhibitors (JAKi); tofacitinib, baricitinib, and upadacitinib [5].

JAKs are intracellular tyrosine kinases that act as key mediators of signal transduction for a range of cytokines, many of which are key drivers of inflammation in RA [6]. In mammals, the JAK family comprises of four members: JAK1, JAK2, JAK3, and Tyk2 (tyrosine kinase 2). Tofacitinib was the first JAKi approved and reimbursed in Australia for the treatment of patients with RA in 2015 and is an oral, small-molecule inhibitor with selectivity towards JAK1 and JAK3 and to a lesser extent JAK2 [6].

At the time of the study, the European League Against Rheumatism (EULAR) recommendations for the treatment of RA suggest that bDMARDs and tsDMARDs should be taken in combination with a csDMARD, most commonly methotrexate (MTX). For patients in which comedication with a csDMARD is contraindicated, the EULAR guidelines suggest that IL6is and tsDMARDs may have some advantages versus the other bDMARDs [7]. Despite these recommendations, it is estimated that one-third of patients prescribed a b/tsDMARD take it as monotherapy [8]. This may be due to a number of reasons including contraindication, unresponsive, or intolerance to the csDMARD or non-adherence to the csDMARD, especially when administered orally [9, 10].

Tofacitinib, similar to the other JAKis, is indicated for use alone or in combination with csDMARDs for the treatment of RA. Theoretically, since JAKi are synthetic and not bDMARDs, they do not provoke an anti-drug antibody response and as such concomitant treatment with MTX should not be necessary [6]. Real-world evidence on the relative efficacy and usage of tofacitinib and bDMARDs in combination with csDMARDs or as monotherapy is limited. Our group recently published a study on the real-world persistence and usage patterns of tofacitinib for the treatment of RA in Australia [11]. This study uses the same cohort from the Optimizing Patient outcome in Australian rheumatoLogy (OPAL) dataset to provide real-world evidence regarding clinical effectiveness, and treatment persistence among patients with RA being treated with monotherapy or combination therapy tofacitinib or bDMARDs.

## Methods

### Study design and setting

Our retrospective, non-interventional cohort study describing the clinical effectiveness, treatment persistence, and treatment patterns in patients prescribed tofacitinib or bDMARDs was recently published [11]. This current report is a post-hoc analysis of that same study cohort describing the effect monotherapy or concomitant csDMARD therapy has on the clinical effectiveness, treatment persistence, and treatment patterns in patients prescribed tofacitinib or bDMARDs. Data were extracted from the Australian OPAL dataset derived from 42 rheumatology clinics around Australia. The OPAL dataset collects information from individual clinicians’ servers entered during routine clinical consultations into purpose-built worksheets in Audit4 software (Software4Specialists, Australia), which also serves as the patient’s medical record [12]. All data extracted was de-identified for patient, clinic, and clinician and exported from each of the OPAL member’s local server, aggregated across all sites, and analyzed. The activities of OPAL Rheumatology Ltd. have received overarching ethics approval from the University of New South Wales (UNSW) Human Research Ethics Committee (HREC), based on a patient opt-out arrangement (HC17799). This research protocol was approved by the UNSW HREC (HC17221).

### Patient population and eligibility criteria

Patients were included if they were registered in the OPAL dataset, had a clinical diagnosis of RA as assessed by treating physician, and were between 18 and 94 years of age. Patients initiated treatment with tofacitinib or a bDMARD, had at least 1 year of follow-up between March 2015 and September 2018. Patients were included if their monotherapy or combination therapy status could be determined. The bDMARDs approved for use in Australia that were included in this study were abatacept, adalimumab, anakinra, certolizumab pegol, etanercept, golimumab, infliximab, rituximab, and tocilizumab. Tofacitinib was the only tsDMARD included in the study as no other tsDMARDs were approved in Australia at the commencement of the study window. Patients who had no visit data recorded and patients who had missing start dates for tofacitinib or bDMARD treatment were not included. Patients with a diagnosis of any autoimmune rheumatic disease or inflammatory bowel disease except for RA were excluded.

### Statistical and analytical assessment

For full details of the methodology, please see original report by Bird et al. [11]. In brief, the primary exposure of interest was an initial prescription for tofacitinib or a bDMARD identified during the sample selection window. Patients that had received a prescription of tofacitinib during the sample selection window were considered part of the “tofacitinib group” even if they had also been prescribed a bDMARD. All other patients were assigned to the bDMARD group. Patients were followed up for a minimum period of 1 year from their index date. Treatment effectiveness was evaluated from baseline (index date) to 18 months using Disease Activity Score-28 with C-reactive protein (CRP) and Simplified Disease Activity Index (SDAI) and Clinical Disease Activity Index (CDAI) measures. Treatment persistence with the index b/tsDMARD was defined as the time (in consecutive days) from the date of treatment initiation until the date of treatment discontinuation. Treatment duration of the index b/tsDMARD was estimated using Kaplan–Meier methods. All analyses were split by monotherapy or combination csDMARD status. The combination therapy group included patients receiving MTX, MTX plus csDMARD and csDMARD excluding MTX.

### Propensity score matching

In order to address the observational nature of the data, propensity score matching was planned between the tofacitinib and bDMARD groups and described in full in Bird et al. [11]. In brief, the propensity score was the conditional probability of receiving treatment (e.g., tofacitinib versus other biologic agent), which was estimated using logistic regression. Covariates included age, sex, and selected baseline (where baseline is the index date) treatment combinations. The baseline treatment combination covariates were methotrexate monotherapy, methotrexate in combination with other csDMARD(s), csDMARD(s) other than methotrexate and neither methotrexate nor other csDMARD(s) (bDMARD monotherapy). Propensity score matching was determined on a ratio of one tofacitinib user to two bDMARD users (1:2) using a caliper width of 0.20.

### Study size

As reported in the original study report data were extracted for 652 patients administered tofacitinib (planned sample size of 500) and 2158 patients administered bDMARDs (planned sample size 2500) [11]. Although the bDMARD sample size was smaller than planned, this sample size still ensured clinically acceptable precision in the estimates of treatment patterns and clinical effectiveness.

## Results

From March 2015 to September 2018, 2810 patients initiated tofacitinib or bDMARDs and met the study selection criteria. Five patients (three in the bDMARD group and two in the tofacitinib group) were excluded because their monotherapy or combination therapy status could not be determined, leaving 2805 patients included in the current analysis. Patient demographics for monotherapy and combination therapy (bDMARD and tofacitinib combined) matched populations are reported in Table 1.

**Table 1:** Patient demographics and disease characteristics at index for the monotherapy and combination propensity score matched population

	Monotherapy	Combination therapy
*N* (%)	846	1104
Age at index (years) Mean (SD)	62.1 (12.6)	60.0 (13.2)
Gender, *n* (% of column) Female	678 (80.1%)	906 (82.1%)
Disease duration in months, median	132.3 [*N* = 493]	99.7 [*N* = 736]
Line of therapy		
1st line	534 (63.1%)	735 (66.6%)
2nd line	309 (36.5%)	362 (32.8%)
3rd line	3 (0.4%)	7 (0.6%)
DAS28-CRP, *n* (% of column)	Rem: 76 (23.8%) Low: 36 (11.3%) Mod: 11 (34.8%) High: 96 (30.1%)	Rem: 66 (12.5%) Low: 38 (7.2%) Mod: 183 (34.7%) High: 240 (45.5%)
CDAI, *n* (% of column)	Rem: 34 (10.7%) Low: 74 (23.3%) Mod: 79 (24.8%) High: 131 (41.2%)	Rem: 23 (4.4%) Low: 61 (11.7%) Mod: 130 (24.9%) High: 309 (59.1%)
TJC28, mean (SD)	6.9 (7.6) [*N* = 482]	10.3 (8.8) [*N* = 703]
SJC28, mean (SD)	6.8 (7.5) [*N* = 482]	10.1 (8.6) [*N* = 703]
RAPID3, mean (SD)	3.3 (2.7) [*N* = 60]	4.1 (2.4) [*N* = 120]

In the overall population, 2155 patients received index treatment with bDMARDs of which 33.4% (*n* = 720) were treated with monotherapy and 650 patients received index treatment with tofacitinib of which 43.4% (*n* = 282) were treated as monotherapy.

In the bDMARD and tofacitinib monotherapy and combination therapy matched populations, there were 1300 bDMARD initiators (564 treated with monotherapy) and 650 tofacitinib initiators (282 of which were treated with monotherapy). Patient demographics for the matched population of bDMARDs and tofacitinib split by monotherapy and combination therapy are reported in Table 2.

**Table 2:** Patient demographics and disease characteristics at index for the propensity score matched population of bDMARD and tofacitinib monotherapy and combination therapy

	Monotherapy		Combination therapy	
	bDMARD	Tofacitinib	bDMARD	Tofacitinib
*N* (%)	564	282	736	368
Age at index (years) Mean (SD)	62.1 (12.8)	62.0 (12.2)	59.8 (13.3)	60.2 (13.0)
Gender, *n* (% of column) Female	452 (80.1%)	226 (80.1%)	604 (82.1%)	302 (82.1%)
Disease duration in months, median	125.4 [*N* = 323]	138.5 [*N* = 170]	90.9 [*N* = 465]	113.1 [*N* = 241]
Line of therapy				
1st line	393 (69.7%)	141 (50.0%)	530 (72.0%)	205 (55.7%)
2nd line	169 (30.0%)	140 (49.6%)	203 (27.6%)	159 (43.2%)
3rd line	2 (0.4%)	1 (0.4%)	2 (0.4%)	4 (1.1%)
Disease status				
DAS28-CRP, *n* (% of column)	Rem: 52 (25.1%) Low: 24 (11.6%) Mod: 71 (34.3%) High: 60 (29.0%)	Rem: 24 (21.4%) Low: 12 (10.7%) Mod: 40 (35.7%) High: 36 (32.1%)	Rem: 36 (10.7%) Low: 24 (7.2%) Mod: 114 (34.0%) High: 161 (48.1%)	Rem: 30 (15.6%) Low: 14 (7.3%) Mod: 69 (35.9%) High: 79 (41.1%)
CDAI, *n* (% of column)	Rem: 25 (12.4%) Low: 46 (22.8%) Mod: 53 (26.2%) High: 78 (38.6%)	Rem: 9 (7.8%) Low: 28 (24.1%) Mod: 26 (22.4%) High: 53 (45.7%)	Rem: 12 (3.6%) Low: 36 (10.9%) Mod: 78 (23.6%) High: 205(61.9%)	Rem: 11 (5.7%) Low: 25 (13.0%) Mod: 52 (27.1%) High: 104 (54.2%)
TJC28, mean (SD)	6.6 (7.5) [*N* = 314]	7.3 (7.7) [*N* = 168]	10.9 (8.9) [*N* = 469]	9.2 (8.5) [*N* = 234]
SJC28, mean (SD)	6.7 (7.6) [*N* = 314]	6.9 (7.6) [*N* = 168]	10.6 (8.8) [*N* = 469]	9.1 (8.3) [*N* = 234]

At index in the matched monotherapy population, age, gender, and the disease activity status were similar in the bDMARD and tofacitinib groups with 29.0% and 32.41% of patients classified as DAS28-CRP high disease activity, respectively. The median disease duration however differed slightly with a disease duration of 125.4 (95%CI 19.4 to 409) months in the bDMARD treated group and 138.4 (95%CI 32.5 to 413.2) months in the tofacitinib treated group. The monotherapy bDMARD group had a higher percentage of patients receiving first line therapy (69.7%) compared to the monotherapy tofacitinib group (50.0%).

In the matched combination therapy population at index, 48.1% and 41.1% of patients were in DAS28-CRP high disease activity in the bDMARD and tofacitinib groups respectively, and the median disease duration was 90.9 (95%CI 11.5 to 379.6) months for the bDMARD group and 113.1 (95%CI 12.5 to 360.2) months for the tofacitinib group. Similar to the monotherapy bDMARD and tofacitinib groups, the combination therapy bDMARD group had a higher percentage of patients receiving first line therapy (72%) compared to the combination therapy tofacitinib group (55.7%).

### Treatment effectiveness and persistence of monotherapy and combination b/tsDMARD therapy

**Fig. 1:**
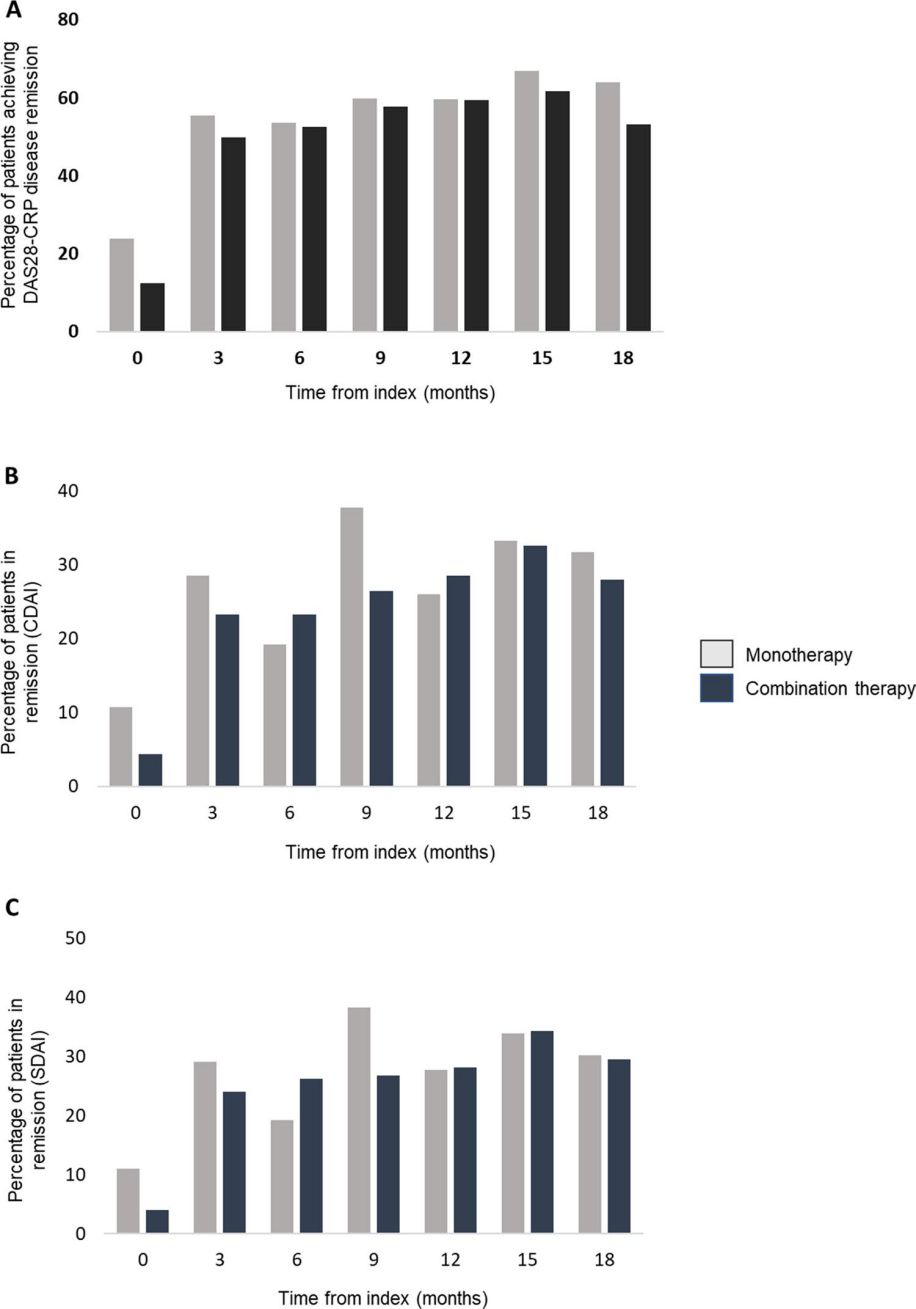
Percentage of patients achieving **A** DAS28-CRP remission, **B** CDAI remission, and **C** SDAI remission in the monotherapy and combination therapy propensity score matched population

The percentage of patients achieving DAS28-CRP, CDAI, and SDAI disease remission in the matched monotherapy and combination therapy groups at 3, 6, 9, 12, and 18 months after index treatment is shown in Fig. 1. At 18 months, 63.9%, 31.7%, and 30.2% of patients treated with monotherapy bDMARD or tofacitinib achieved DAS28-CRP, CDAI, and SDAI disease remission, respectively. In the combination therapy group after 18 months, 53.2%, 29.5%, and 28% of patients achieved DAS28-CRP, CDAI, and SDAI disease remission, respectively. In the matched population, the median persistence was 34.5 months (95% CI 30.3 to “not reached”) for patients treated with monotherapy b/tsDMARDs and 32.7 months (95% CI 28.8 to “not reached”) for patients treated with combination therapy b/tsDMARDs (data not shown).

### DAS28-CRP disease remission in bDMARD and tofacitinib matched monotherapy and combination therapy groups

**Fig. 2:**
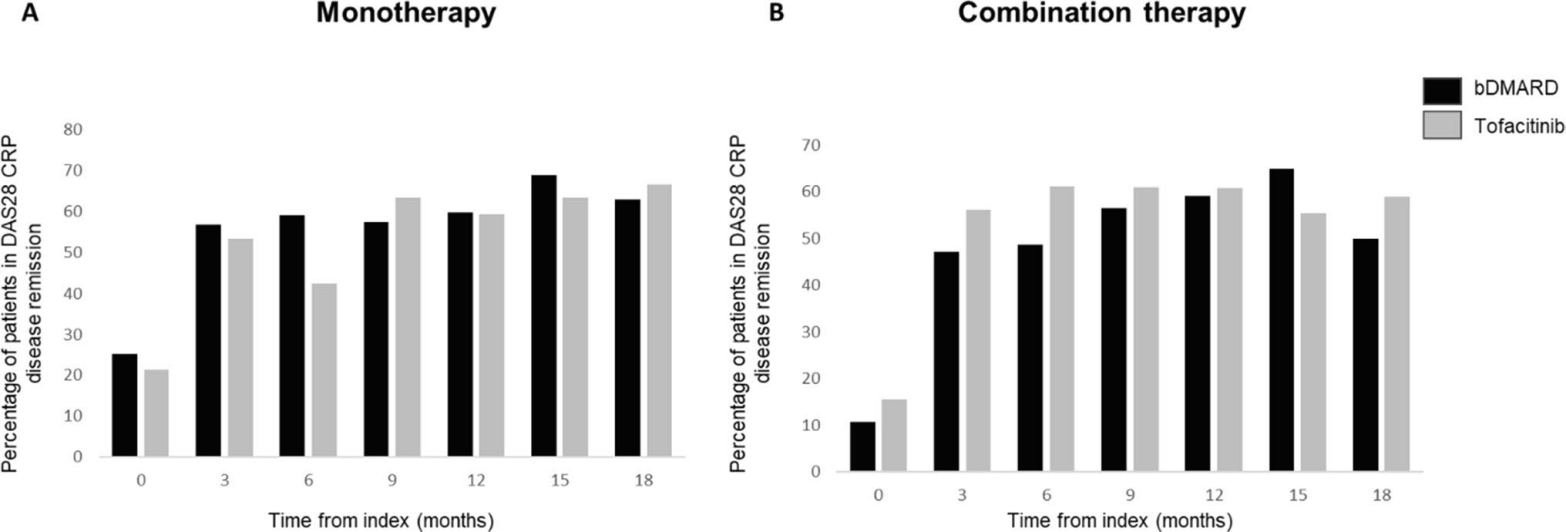
Percentage of patients in DAS28-CRP disease remission in the **A** bDMARD and tofacitinib monotherapy population and **B** the bDMARD and tofacitinib combination therapy population

The percentage of patients in DAS-28 CRP disease remission in the bDMARD and tofacitinib monotherapy and combination therapy matched populations is shown in Fig. 2. At index, 25.1% and 21.4% of patients treated with monotherapy bDMARD or monotherapy tofacitinib respectively were in DAS-28 CRP disease remission (Fig. 2A). After 3 months of treatment, 56.8% and 53.4% were in DAS-28 CRP disease remission and after 18 months of treatment, 62.9% and 66.7% had reached DAS-28 CRP disease remission in the bDMARD monotherapy and tofacitinib monotherapy groups, respectively. There were no significant differences in remission rates between the monotherapy bDMARD and monotherapy tofacitinib groups at any of these time points.

At index, 10.7% and 15.6% of patients treated with combination bDMARD or combination tofacitinib respectively were in DAS-28 CRP disease remission. After 3 months of treatment, 47.2% and 56.0% had achieved DAS-28 CRP disease remission and after 18 months of treatment, 50.0% and 58.9% had reached DAS-28 CRP disease remission in the bDMARD combination and tofacitinib combination therapy groups, respectively (Fig. 2B). There were no significant differences in remission rates between the combination therapy bDMARD and combination therapy tofacitinib groups at any of these time points.

### CDAI and SDAI remission in bDMARD and tofacitinib matched monotherapy and combination therapy groups

The percentage of patients achieving CDAI and SDAI disease remission for both the bDMARD and tofacitinib monotherapy and combination therapy propensity score matched populations is shown in Online Resource 1. The percentage of bDMARD monotherapy patients achieving CDAI and SDAI remission at 18 months was 33.8% and 31.4%, respectively. The percentage of tofacitinib monotherapy patients achieving CDAI and SDAI disease remission after 18 months was 25.9% and 26.9% respectively (Online Resource 1A and 1B). In the combination therapy propensity score matched populations, the percentage of patients achieving CDAI and SDAI disease remission in the bDMARD group was 25.5% and 27.7% respectively, while in the tofacitinib combination therapy group 32.7% achieved CDAI and 32.7% SDAI disease remission at 18 months (not statistically significant) (Online Resource 1C and 1D).

### Treatment persistence

**Fig. 3:**
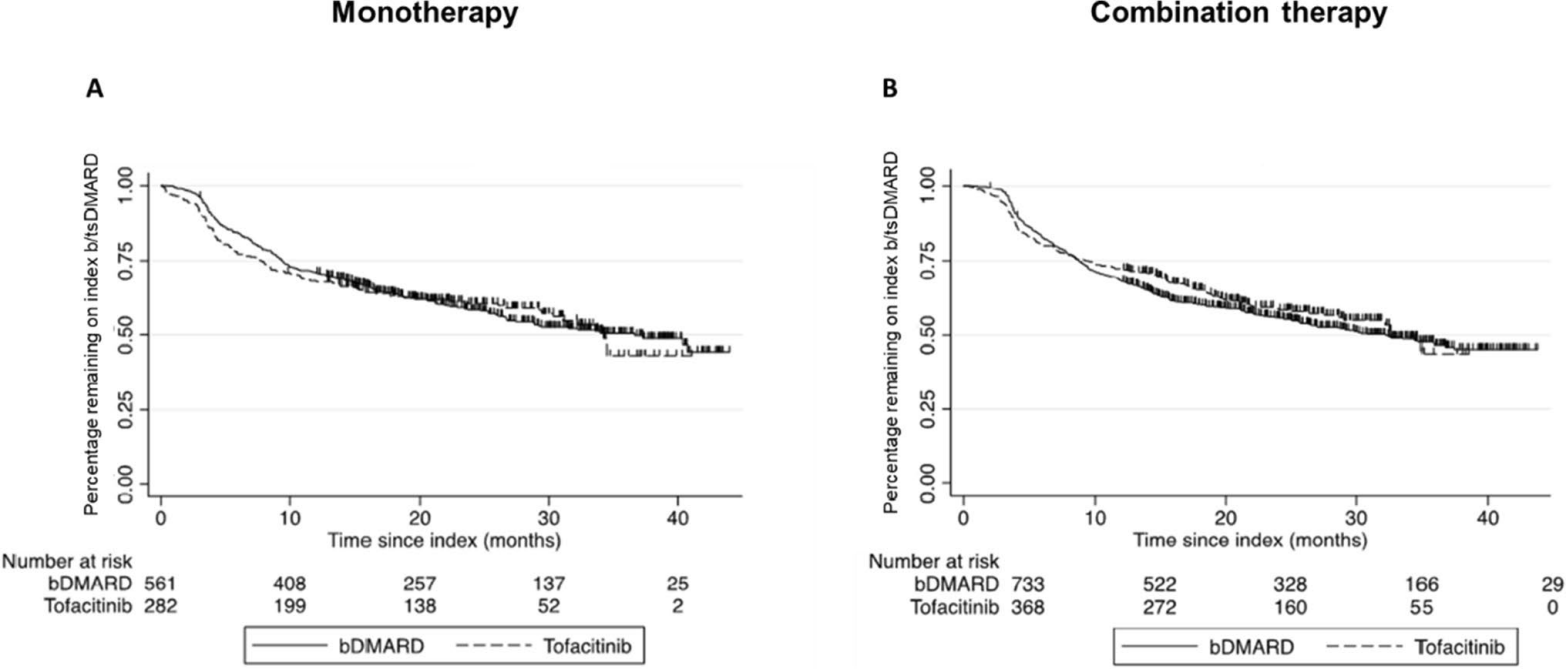
Treatment persistence of matched population of **A** bDMARD and tofacitinib monotherapy and **B** bDMARD and tofacitinib combination therapy

In the monotherapy matched population, the median persistence for patients prescribed bDMARDs was 36.7 months (95% CI 27.4 to “not reached”) and 34.2 months (95%CI 30.3 to “not reached”) for patients prescribed tofacitinib (Fig. 3A). In the combination therapy matched population, the median persistence for patients prescribed bDMARDs was 32.2 months (95% CI 25.7 to 34.4) and 32.7 months (95%CI 28.7 to “not reached”) for patients prescribed tofacitinib (Fig. 3B).

### Reasons for discontinuation

The most common reasons for discontinuing monotherapy or combination therapy bDMARD or tofacitinib are provided in Online Resource 2. In the monotherapy matched population, the most common reasons for discontinuation were better alternative, lack of efficacy, and adverse reaction. In the combination matched groups, better alternative, lack of efficacy, and lack of efficacy due to secondary failure were the most common reasons recorded. No adverse events were reported in more than 5% of the bDMARD or tofacitinib overall or matched populations.

### Treatment patterns

Monotherapy and csDMARD treatment patterns at initiation and at 74 to 104 weeks of follow-up for the overall monotherapy and combination therapy treatment groups are shown in Online Resource 3. At 74 to 104 weeks follow-up, 12.3% of the bDMARD and 7.9% of the tofacitinib monotherapy population had moved to combination therapy with a csDMARD. Conversely for the combination therapy group at 74 to 104 weeks of follow-up, 12.9% of bDMARD and 17.2% of tofacitinib treated patients were on monotherapy treatment. It is important to note that this data is limited to patients who remain on their index b/tsDMARD at the time of follow-up.

## Discussion

This study explored the real-world clinical effectiveness and treatment persistence among patients with RA treated with monotherapy and combination tofacitinib and bDMARD therapy. In both the monotherapy and combination therapy matched groups the age, gender, and disease activity status of the bDMARD and tofacitinib groups were similar. Patients initiating tofacitinib however had longer disease duration in both monotherapy and combination therapy arms, which has been reported previously in this patient cohort [11] as well as in the US Corrona dataset [13]. The bDMARD group (both monotherapy and combination therapy) also had a higher percentage of patients on their first line bDMARD compared to tofacitinib.

Co-administration of a b/tsDMARD with a csDMARD such as methotrexate has documented advantages in some cohorts which include augmenting the reduction in radiographic damage, attenuating anti-drug antibodies against bDMARDs, as well as increasing the bioavailability of some bDMARDs [10]. Co-morbidity associated with combination therapy is also well documented, and therefore physicians and patients work together to formulate a balanced approach in each individual. In Australia, the majority of b/tsDMARDs are approved for the treatment of RA as monotherapy or in combination with csDMARDs. Although international guidelines recommend that b/tsDMARDs are used in combination with a csDMARD, up to one-third of patients are treated with monotherapy [8]. In our cohort, one-third (33.4%) of patients treated with a bDMARD were on monotherapy; however, 43.4% of patients treated with tofacitinib were treated as monotherapy. The higher percentage of patients treated with tofacitinib monotherapy compared to bDMARD monotherapy may be due to data demonstrating greater efficacy of the TNFis when used in combination therapy versus monotherapy [14]. However, in the Oral Rheumatoid Arthritis triaL (ORAL) Strategy study tofacitinib monotherapy did not show non-inferiority to either tofacitinib and methotrexate or adalimumab and methotrexate, indicating that similar to the TNFi’s, patients will typically respond better to tofacitinib if combined with methotrexate [15].

In both the overall and matched populations, patients prescribed bDMARDs or tofacitinib in combination with csDMARD(s) had higher disease activity at index than those prescribed monotherapy bDMARD or tofacitinib. This may suggest that rheumatologists treating this patient cohort are more likely to use combination therapy for patients with higher disease activity scores. This is supported by literature demonstrating that, in some cohorts, combination therapy improves clinical response, functional outcome and delays radiographic progression compared with monotherapy. As such, a “step-down” strategy has been proposed to offer a better chance for quicker, more effective suppression of inflammation versus a “step up” strategy [reviewed in 14].

In the current study, both monotherapy and combination therapy treatment regimens resulted in an increased number of patients achieving DAS28-CRP, CDAI, and SDAI disease activity remission after 3 months, which was maintained out to 18 months. Although the percentage of patients achieving CDAI and SDAI remission at 18 months was similar for both the monotherapy and combination therapy matched groups, the percentage of patients achieving DAS28-CRP remission at 18 months was higher for the monotherapy group compared with the combination therapy group (63.9% vs. 53.2%, respectively). This may be due to differences in the characteristics between the monotherapy and combination therapy populations such as a higher percentage of patients in remission and a lower percentage of patients in high disease activity in the monotherapy group at index. Lower disease activity has been shown in the GLADAR (Grupo Latino Americano De estudio de la Artritis Reumatoide) cohort to consistently predict remission and LDA in patients with RA after 1 year and 2 years follow-up [16].

A numerically higher percentage of patients treated with monotherapy bDMARD achieved DAS28-CRP remission or LDA at 6, 9, and 12 months after initiation of their index bDMARD as compared to monotherapy tofacitinib; however, this was not found to be statistically significant. At 15 and 18 months however, the percentage of patients in DAS28-CRP remission or LDA was similar for both monotherapy tofacitinib and monotherapy bDMARD patients. The tofacitinib monotherapy group had longer disease duration and a lower percentage of first-line patients than the bDMARD monotherapy population which may explain the slight differences between the bDMARD and tofacitinib monotherapy groups at the earlier time points. There were no differences in the remission rates seen between the tofacitinib combination therapy and bDMARD combination therapy treated patients.

Similar real-world persistence rates have been reported for tofacitinib and the bDMARDs [11, 17]; however, to the best of our knowledge, real-world persistence with monotherapy tofacitinib and bDMARDs and combination therapy tofacitinib and bDMARDs has not been previously reported. While concomitant use of MTX with bDMARDs can prolong treatment durability through the attenuation of anti-drug antibodies [18], in this study, there was no difference in the median persistence rates for monotherapy bDMARD when compared with monotherapy tofacitinib treatment or combination therapy bDMARD and combination therapy tofacitinib.

It is also interesting to note that in this patient cohort the majority of patients that initiated b/tsDMARD as monotherapy (and were still on their index treatment at time of follow-up), remained on monotherapy at 74 to 104 weeks follow-up. For those that initiated as combination therapy, at 74 to 104 weeks later almost 13% of bDMARD treated patients moved to monotherapy, whereas 17% of tofacitinib treated patients transitioned to monotherapy.

Our study suggests that both monotherapy and combination therapy use of tofacitinib is an effective treatment for patients with RA with comparable treatment effectiveness and persistence to bDMARDs. Further analysis of emerging real-world data of the approved JAKis is required to provide further evidence of their sustained efficacy and safety in patients with RA.

## Limitations

This was a retrospective study of observational data and as such there are a number of limitations including a large proportion of missing data. The variables, sample size, and study duration were selected in order to minimize the impact of this. There may have also been confounding due to differences in the duration of disease at the commencement of the index b/tsDMARD with those treated with tofacitinib had longer disease duration at index compared to the bDMARD group.

## Supplementary Information

Below is the link to the electronic supplementary material.

Supplementary file1 (PDF 161 KB)

Supplementary file2 (PDF 96.4 KB)

Supplementary file3 (PDF 101 KB)

## Data Availability

Not applicable.
