# Supplementary material for: Real-world evaluation of effectiveness, persistence, and usage patterns of monotherapy and combination therapy tofacitinib in treatment of rheumatoid arthritis in Australia
Source: Clin Rheumatol. 2021 Aug 9;41(1):53–62. doi: 10.1007/s10067-021-05853-x (PMC8724080; doi:10.1007/s10067-021-05853-x)
Supplement: Supplementary file 1 — Supplementary file1 (PDF 161 KB) [file 10067_2021_5853_MOESM1_ESM.pdf]

Real-world evaluation of effectiveness, persistence, and usage patterns of monotherapy and combination therapy tofacitinib in treatment of rheumatoid arthritis in Australia

Paul Bird[1], Geoffrey Littlejohn[2,3], Belinda Butcher[1,4], Tegan Smith[2], Catherine O'Sullivan[2], David Witcombe[5] & Hedley Griffiths[2,6]

1. University of New South Wales, Kensington, New South Wales, Australia
2. OPAL Rheumatology Ltd, Sydney, New South Wales, Australia
3. Monash University, Clayton, Victoria, Australia
4. WriteSource Medical Pty Ltd, Lane Cove, New South Wales, Australia
5. Pfizer Australia, Sydney, New South Wales, Australia
6. Barwon Rheumatology Service, Geelong, Victoria, Australia

Corresponding Author:

Name: Catherine O'Sullivan

Email: cath.osullivan@opalrheumatology.com.au

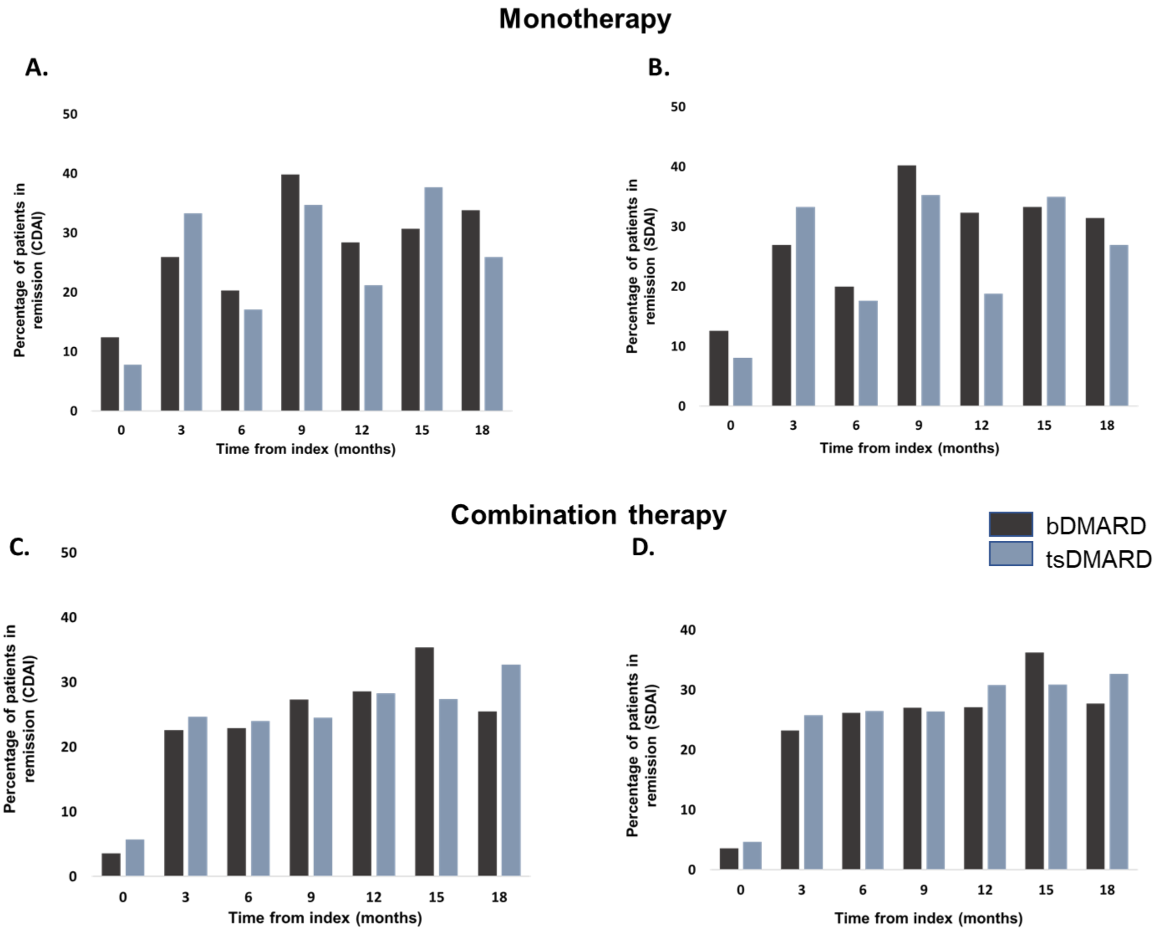

Online Resource 1. Percentage of patients achieving A) CDAI and B) SDAI disease remission in the bDMARD and tofacitinib monotherapy propensity score matched population and C) CDAI and D) SDAI disease remission in the bDMARD and tofacitinib combination therapy propensity score matched population
