# Supplementary material for: Real-world evaluation of effectiveness, persistence, and usage patterns of monotherapy and combination therapy tofacitinib in treatment of rheumatoid arthritis in Australia
Source: Clin Rheumatol. 2021 Aug 9;41(1):53–62. doi: 10.1007/s10067-021-05853-x (PMC8724080; doi:10.1007/s10067-021-05853-x)
Supplement: Supplementary file 2 — Supplementary file2 (PDF 96.4 KB) [file 10067_2021_5853_MOESM2_ESM.pdf]

Real-world evaluation of effectiveness, persistence, and usage patterns of monotherapy and combination therapy tofacitinib in treatment of rheumatoid arthritis in Australia

Paul Bird[1], Geoffrey Littlejohn[2,3], Belinda Butcher[1,4], Tegan Smith[2], Catherine O'Sullivan[2], David Witcombe[5] & Hedley Griffiths[2,6]

1. University of New South Wales, Kensington, New South Wales, Australia
2. OPAL Rheumatology Ltd, Sydney, New South Wales, Australia
3. Monash University, Clayton, Victoria, Australia
4. WriteSource Medical Pty Ltd, Lane Cove, New South Wales, Australia
5. Pfizer Australia, Sydney, New South Wales, Australia
6. Barwon Rheumatology Service, Geelong, Victoria, Australia

Corresponding Author:

Name: Catherine O'Sullivan

Email: cath.osullivan@opalrheumatology.com.au

Online Resource 2. Main reasons given for discontinuation of bDMARD and tofacitinib.

Reasons for discontinuation	Monotherapy		Combination therapy	
	bDMARD	Tofacitinib	bDMARD	Tofacitinib
Better alternative	20.4%<br>(n = 168/823)	14.8%<br>(n = 40/271)	14.5%<br>(n = 141/972)	5.9%<br>(n= 23/388)
Lack of efficacy	14.8%<br>(n = 122/823)	24.7%<br>(n = 67/271)	15.3%<br>(n = 149/972),	12.1%<br>(n= 87/388)
Adverse reaction	12.8%<br>(n = 105/823)	14.0%<br>(n = 38/271)	11.4%<br>(n = 111/973)	10.3%<br>(n = 40/388)
Lack of efficacy, secondary failure	8.7%<br>(n =72/823)	9.6%<br>(n = 26/271)	11.3%<br>(n = 110/972)	22.4%<br>(n= 87/388)
