# Supplementary material for: Real-world evaluation of effectiveness, persistence, and usage patterns of monotherapy and combination therapy tofacitinib in treatment of rheumatoid arthritis in Australia
Source: Clin Rheumatol. 2021 Aug 9;41(1):53–62. doi: 10.1007/s10067-021-05853-x (PMC8724080; doi:10.1007/s10067-021-05853-x)
Supplement: Supplementary file 3 — Supplementary file3 (PDF 101 KB) [file 10067_2021_5853_MOESM3_ESM.pdf]

Journal of Clinical Rheumatology

Real-world evaluation of effectiveness, persistence, and usage patterns of monotherapy and combination therapy tofacitinib in treatment of rheumatoid arthritis in Australia

Paul Bird[1], Geoffrey Littlejohn[2,3], Belinda Butcher[1,4], Tegan Smith[2], Catherine O'Sullivan[2], David Witcombe[5] & Hedley Griffiths[2,6]

1. University of New South Wales, Kensington, New South Wales, Australia
2. OPAL Rheumatology Ltd, Sydney, New South Wales, Australia
3. Monash University, Clayton, Victoria, Australia
4. WriteSource Medical Pty Ltd, Lane Cove, New South Wales, Australia
5. Pfizer Australia, Sydney, New South Wales, Australia
6. Barwon Rheumatology Service, Geelong, Victoria, Australia

Corresponding Author:

Name: Catherine O'Sullivan

Email: cath.osullivan@opalrheumatology.com.au

Online Resource 3. Monotherapy and combination therapy treatment patterns at treatment initiation and at 74 to 104 weeks follow-up in the monotherapy and combination therapy overall populations.

	Monotherapy (overall population)				Combination therapy (overall population)			
	Index		74 to < 104 weeks follow-up		Index		74 to < 104 weeks follow-up	
	bDMARD (N = 720) n (%)	TOF (N = 282) n (%)	bDMARD (N = 463) n (%)	TOF (N = 181) n (%)	bDMARD (N = 1435) n (%)	TOF (N =368) n (%)	bDMARD (N = 866) n (%)	TOF (N = 244) n (%)
MTX + other csDMARD	0 (0%)	0 (0%)	2 (0.4%)	0 (0.0%)	589 (41.0%)	146 (39.7%)	286 (30.9%)	78 (32.0%)
MTX only	0 (0%)	0 (0%)	31 (6.7%)	7 (3.9%)	556 (38.7%)	150 (40.8%)	355 (41.0%)	79 (32.4%)
csDMARD (excl. MTX)	0 (0%)	0 (0%)	24 (5.2%)	7 (3.9%)	290 (19.6%)	72 (19.6%)	131 (15.1%)	45 (18.4%)
bDMARD monotherapy	720 (100%)	282 (100%)	406 (87.7%)	167 (92.1%)	0 (0%)	0 (0%)	112 (12.9%)	42 (17.2%)

This is limited to patients who remain on their index b/tsDMARD at the time
